# Advanced Genetic Testing Comes to the Pain Clinic to Make a Diagnosis of Paroxysmal Extreme Pain Disorder

**DOI:** 10.1155/2016/9212369

**Published:** 2016-07-21

**Authors:** Ashley Cannon, Svetlana Kurklinsky, Kimberly J. Guthrie, Douglas L. Riegert-Johnson

**Affiliations:** ^1^The Department of Medical Genetics, Mayo Clinic, Jacksonville, FL 32224, USA; ^2^Center for Individualized Medicine, Mayo Clinic, Jacksonville, FL 32224, USA; ^3^Department of Pain Medicine, Mayo Clinic, Jacksonville, FL 32224, USA

## Abstract

*Objective*. To describe the use of an advanced genetic testing technique, whole exome sequencing, to diagnose a patient and their family with a* SCN9A *channelopathy.* Setting*. Academic tertiary care center.* Design*. Case report.* Case Report*. A 61-year-old female with a history of acute facial pain, chronic pain, fibromyalgia, and constipation was found to have a gain of function* SCN9A* mutation by whole exome sequencing. This mutation resulted in an* SCN9A* channelopathy that is most consistent with a diagnosis of paroxysmal extreme pain disorder. In addition to the patient being diagnosed, four siblings have a clinical diagnosis of* SCN9A *channelopathy as they have consistent symptoms and a sister with a known mutation. For treatment, gabapentin was ineffective and carbamazepine was not tolerated. Nontraditional therapies improved symptoms and constipation resolved with pelvic floor retraining with biofeedback.* Conclusion*. Patients with a personal and family history of chronic pain may benefit from a referral to Medical Genetics. Pelvic floor retraining with biofeedback should be considered for patients with a* SCN9A* channelopathy and constipation.

## 1. Introduction

The* SCN9A *gene encodes the Na_v_1.7 voltage-gated sodium channel. Mutations in the* SCN9A *gene are the cause of a heterogeneous channelopathy pain syndrome. The Na_v_1.7 channel is highly expressed in peripheral somatic and visceral sensory neurons, the nociceptive neurons at dorsal root ganglion (DRG), trigeminal ganglion, olfactory sensory neurons, and sympathetic ganglion [1–6]. Na_v_1.7 amplifies small subthreshold depolarizations [1], regulating excitability of the membrane potential and positioning this sodium channel as a molecular gatekeeper for pain. Heterozygous (monoallelic) gain of function* SCN9A *mutations is associated with multiple phenotypes including small nerve fiber neuropathy (SFN), inherited erythromelalgia (IEM), paroxysmal extreme pain disorder (PEPD) (Table 1), and a novel syndrome of pain dysautonomia [7]. Distinctions between syndromes are made based on presenting symptoms because the same mutation can vary in severity of symptoms and manifest as a different syndrome classification for reasons that are not currently understood. Even in siblings the I228M variant of sodium channel Na_v_1.7 has been shown to represent itself as both IEM and SFN [8]. The difference in presentation is possibly due to the different patterns of modifier genes, epigenetics, or posttranslational modification, for example, glycosylation [9].

Besides gain of function mutations, biallelic damaging* SCN9A *mutations are associated with congenital insensitivity to pain. A large percentage of small nerve fiber neuropathies have an unknown etiology [10, 11].

Several new technologies, all referred to as next-generation sequencing (NGS), have decreased the cost while increasing the speed of gene sequencing. Using NGS it is now feasible to sequence the coding sequences of all 20,000 genes referred to as the exome. Whole exome sequencing (WES) has been clinically available since 2011 and is now offered by several laboratories. Eighty percent of known disease-causing mutations are located in the exome, but the exome represents only 1% of the total genome. Even this 1% of the genome constitutes a vast amount of data, about 30,000,000 base pairs of DNA.

Next-generation sequencing facilitates the identification of individuals with Mendelian disorders that may be underdiagnosed due to variable or relatively nonspecific clinical findings. In this way, such technology will ultimately impact a greater number of medical specialties including Pain Medicine. We describe a case where NGS was used to make a clinically unsuspected diagnosis of PEPD giving the patient a diagnosis and additional treatment options.

PEPD has been described in less than 500 patients [12–15] predominantly in the UK and Netherlands. Clinical features of PEPD are sudden, painful attacks of the anorectal, ocular, and mandibular areas, as well as tonic nonepileptic seizures, flushing, watering of eyes or nose, hypersalivation, and weakness related to the site of pain that can last from hours to days [12, 16–18]. Triggers of the pain can be trauma, childbirth, defecation, cold, and nonphysical triggers such as strong emotions. We describe the use of NGS to make the diagnosis of PEPD and how a mutation reported previously to cause SFN can cause PEPD [14] and give our recommendations for Pain Clinic referrals to Medical Genetics.

## 2. Case Report

A 61-year-old female of Puerto Rican ancestry was evaluated in Medical Genetics for seemingly separate chronic pain syndromes. She had previously been seen in Family Practice, Gastroenterology, Pain Medicine, Neurology, Physical Medicine and Rehabilitation, Gynecology, and Ophthalmology departments.

**Figure 1:**
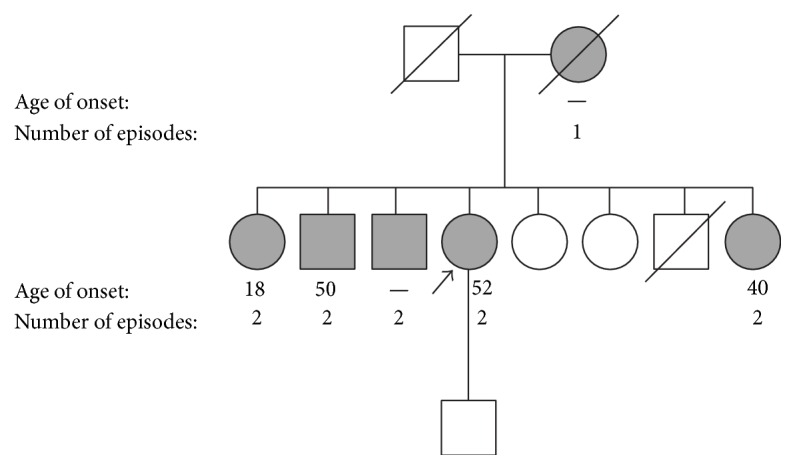
*Pedigree*. The patient is indicated by an arrow and all relatives that exhibited at least one episode of extreme facial pain are in grey. The age of onset and number of episodes are indicated beneath each affected individual.

The patient had attacks of facial pain; preliminary diagnosis of Melkersson-Rosenthal syndrome was made. Melkersson-Rosenthal syndrome is a rare nonhereditary disorder characterized by facial weakness, swelling of the face and lips, and furrowing of the tongue. The patient reported similar symptoms in other family members. As familial Melkersson-Rosenthal syndrome had not been reported in the literature, the patient was referred to Medical Genetics for further evaluation. A detailed family history revealed similar facial pain episodes in the patient's mother and four of seven siblings, suggesting a genetic condition of autosomal dominant inheritance (Figure 1). The age of onset of symptoms in the family ranged from 18 to 52 years.

### 2.1. The Patient Had a Complex Array of Symptoms

The patient had pain involving both sides of the body, both above and below the waistline, including the posterior cervical region. The areas of most severe pain were in the neck, shoulders, both upper extremities, entire back, buttock area, and bilateral lower extremities. Heating pads seem to improve the constant pain and patient preferred a room temperature of 27°C (80°F).

The episodic pain triggers included stressful events (work meetings), defecation, and cold temperatures.

The patient's facial pain symptoms were most consistent with a diagnosis of PEPD. She had two separate episodes of severe facial pain that resulted in hospitalization at the ages of 52 and 61 years. The first episode consisted of severe headache lasting 3 days, right facial swelling, right eye weakness, and forehead paralysis. The second episode consisted of a global thunderclap headache, left orbital eye pain, left facial paralysis with decreased sensation, and right-sided ptosis, in addition to facial swelling and weakness. MR and CT brain imaging were unremarkable.

The facial pain also occurred several times each day and was triggered by cold, smiling, brushing teeth, and chewing, but not by touching the face or food. Paroxysmal type of electrical pain shoots down the right mandible. This may be followed by a pulling sensation in her right cheek and eye. Intensity was typically 5 on a scale of 10, although it may reach 10. The pain was accompanied by dripping lacrimation in the right eye but no facial flushing or sweating (Table 3). The lacrimation was worse when she was eating but she does not describe gustatory sweating (Table 3). Patient's facial pain lasted for several hours. Patient avoids eating cold foods like popsicles and melts ice cream before eating it. Patient also avoids eating in public.

Sinus congestion previously triggered pain, but this mostly resolved after three sinus operations. She denied tear secretion and flushing being triggers for pain (Table 3).

She described often having rectal pain during defecation, consistent with PEPD. This began around the age of 45 and had progressively increased in severity. She described the rectal pain severity to be worse than pain associated with childbirth (10/10); the pain attacks lasted for hours.

She denied having seizures, including the period during childhood; however, patient had several syncope events as an adult.

She reported “fibromyalgia attacks” that occur about twice a month, after stressful events like meetings and cold temperature, which affect the entire body and pain was described as achy and dull; pain lasted for several days. The patient was previously diagnosed with fibromyalgia by Family Medicine at the age of 56 by the American College of Rheumatology 1990 criteria. These findings suggest a complex pain phenotype that consisted of pain attacks akin to PEPD in addition to persistent pain that can be observed in SFN. The patient was also asked about symptoms specific to PEPD.

The patient completed the Neuropathic Pain Scale to evaluate the intensity and characteristics of pain [35]. She described a constant pain that was intense, sharp, deep, and often cold. The intensity was reported as a 5/10 in the coccyx and lower back. She described episodic sharp pain (10/10) in the neck, legs, and back. She reported that the coccyx pain radiates most often to her left leg but sometimes radiates down both legs, lasting for hours. She also described a cold pain (8/10), especially in her feet, even on warm days.

Additionally, the patient completed a small nerve fiber questionnaire (Table 2), which examined the presence and frequency of features associated with SFN such as sweating, diarrhea, constipation, incontinence, dry eyes, dry mouth, dizziness upon standing, palpitations, hot flashes, skin sensitivity, burning feet, sheet intolerance, and restless legs. She reported persistent constipation, dry eyes with occasional diarrhea, and excessive sweating. She denied urination problems, dry mouth, dizziness upon standing, palpitations, hot flashes, sensitive skin, burning feet, sheet intolerance, or restless legs. These findings indicate that the patient exhibits certain features observed in SFN like neuropathic pain, persistent pain, and perhaps some of the autonomic features.

The patient was asked about symptoms of IEM. She did not have severe burning pain in the distal limbs or pain triggered by warmth.

EMG was performed by neurology when patient was 55 and 56 years old for evaluation of Bell's palsy; the first was one was unremarkable, while the second one showed mononeuropathy at the wrist.

### 2.2. Whole Exome Sequencing (WES) Genomic Testing, Diagnosis, and Treatment

“The patient was referred with a diagnosis of familial Melkersson-Rosenthal syndrome. No gene has been associated with Melkersson-Rosenthal syndrome before. The authors considered several strategies to hunt for the Melkersson-Rosenthal gene. The authors could not identify any strong candidate genes for Sanger sequencing. It was decided to use the WES approach of testing about 20,000 genes with one test.”

A sample of the patient's blood was sent to Baylor Medical Genetics Laboratories (Houston, TX) for WES using an Illumina HiSeq platform (San Diego, CA). WES analysis detected a pathogenic heterozygous c.2159T>A (p.I720K) mutation in the* SCN9A *gene. The cost of the WES test was $7000 (USA); the results were available for us after 9 weeks. This patient's testing was paid for by the Mayo Clinic Individualized Medicine Clinic. We estimate the mean out of pocket expense for WES for patients with commercial insurance at $1000 or less.

Treatment options for* SCN9A *channelopathies are suboptimal. Patients with PEPD have experienced some benefit with carbamazepine [13]. This patient was treated with oxcarbazepine 150 mg twice daily. After 6 weeks of oxcarbazepine treatment she developed Stevens-Johnson syndrome, with skin redness and peeling. Her pain had not improved with oxcarbazepine. Oxcarbazepine was stopped and gabapentin was then begun. The gabapentin regimen was 100 mg three times a day (10 days), 300 mg three times a day (10 days), and finally 600 mg three times per day (10 days). Gabapentin was stopped after the patient reported no improvement and that it was too sedating.

The patient was next offered lacosamide. Lacosamide is an adjunctive treatment for partial onset seizures and diabetic neuropathic pain. Lacosamide enhances slow inactivation of Na_v_1.7 and as such may be a valuable choice of treatment. A clinical trial of lacosamide for patients with* SCN9A *mutations has been registered and is recruiting (NCT01911975). The patient declined lacosamide after she was told there was an increased risk of suicidal behavior and ideation (as with all antiepileptic drugs).

The patient reported that nontraditional therapies have been the most effective at treating her pain. Following severe facial pain episodes, her symptoms improved with massage, acupuncture, and cold compresses. Heating pads have improved her constant pain.

The cause of the patient's constipation was diagnosed as pelvic floor dysfunction by anal rectal manometry and MR defecography. The patient described remarkable improvement in constipation after pelvic floor retraining with biofeedback. A physical therapist administered treatment once a week for six weeks (standard regime for our and other centers). Treatment consisted of external therapeutic ultrasound, 5 min of therapeutic exercise, and 30 min of soft tissue mobilization with manual therapy to gluteal muscles, piriformis, coccygeus, ST ligaments, levator ani, and ischiopubic points. At the end of treatment, she was having one bowel movement most days compared to a bowel movement once a month since early childhood.

Return of constipation after pelvic floor retraining with biofeedback occurs in about half of patients at one year [36]. This patient's constipation returned after 4 years and the patient had a second course of pelvic floor retraining with biofeedback. The treatment was again effective and her constipation resolved. We speculate that her pelvic floor dysfunction was a compensatory response to rectal pain during defecation resulting from the* SCN9A* mutation.

## 3. Discussion

### 3.1. Patients with* SCN9A* Mutations Can Present Features of Different Pain Syndromes

Here, we report a patient with an* SCN9A *c.2159T>A mutation that has been reported in one patient before [14]. The patient's clinical presentation is most consistent with PEPD, including severe episodic pain in the facial and anorectal regions that are triggered by stressful events, cold temperatures, and defecation. In addition, she presents with persistent neuropathic pain and autonomic dysfunction, including severe constipation, which is consistent with SFN.

The* SCN9A* c.2159T>A mutation detected in our patient has been reported in a single Dutch patient diagnosed with SFN [14]. This patient experienced severe pain throughout the body with muscle ache before developing the characteristic distal pain of SFN, with burning pain in feet, lower arms, and lower legs, numbness in feet, and hyperhidrosis in feet; the Dutch patient also suffered from hot flashes, skin hyperesthesia, sheet intolerance, restless legs, sweating, diarrhea, micturition, dry eyes and mouth, dizziness, and palpitations [14]. The comparison between the Dutch patient and our patient indicates that even the same* SCN9A *mutation can manifest differently in different patients. Because of the inconsistent relationship between the mutation and the outcome (genotype-phenotype correlation), we and others recommend diagnosis of a specific* SCN9A *channelopathy based on the patient's clinical status.

### 3.2. *SCN9A* and Fibromyalgia

The patient suffered from complex pain symptoms, fibromyalgia, and other symptoms of the central sensitization syndrome like depression and anxiety [37, 38]. Stimulation of C-fibers has been implicated in the mechanism of central sensitization syndrome, mainly through the NMDA receptor's role in the spinal transmission of nociceptive signals, and contributes to inflammatory nociceptive sensitization [39, 40]. Na_v_1.7 channels have been found on the C-fibers where they could be leading to hyperexcitability and promoting signaling of the NMDA receptors. It would be interesting to see if other patients with fibromyalgia and central sensitization syndrome have mutations in* SCN9A *or proteins that support Na_v_1.7 function, like scaffolds of anchoring proteins. Notably, a study of Mexican women demonstrated that an* SCN9A *polymorphism significantly correlated with women diagnosed with fibromyalgia [41]. Because sodium channels located in nociceptors act as molecular gatekeepers for pain stimulation regulating membrane potential, it is possible that fibromyalgia has polygenetic components where sodium channels have an important role.

### 3.3. Pelvic Floor Biofeedback for PEPD

PEPD is classically associated with rectal pain and constipation. This is the first report of pelvic floor biofeedback for PEPD. Given the excellent response our patient had, we recommend pelvic floor biofeedback be considered for PEPD and* SCN9A *channelopathy patients with rectal pain or constipation. The biofeedback treatment should be tailored to the individual patient.

### 3.4. WES and the Importance of Making a Diagnosis

WES is a powerful diagnostic technique that can make a diagnosis in 1 of 4 patients in genetics clinics where traditional means of evaluation have failed to make a diagnosis [42]. In our experience, much of the power of WES comes from not being biased by previous clinical judgments and errors. This is illustrated in this case of the diagnosis of Melkersson-Rosenthal syndrome in patient with a clear family history, although Melkersson-Rosenthal syndrome is not known to be hereditary. Additionally, the reported family history of multiple affected family members in two generations increased the likelihood of identifying a single gene disorder by whole exome sequencing.

### 3.5. The Value of a Diagnosis Cannot Be Overestimated

Although this patient has not had a clear clinical benefit from her diagnosis to date, she has been offered more options (e.g., lacosamide) and may benefit in the future. Individuals attending Medical Genetics clinics often report their primary concern is their children and other family members. The identification of* SCN9A *mutation has benefitted both her son and siblings. The patient's son and siblings can now be tested to determine whether they carry the familial* SCN9A *mutation. Mutation carriers in the family can now have treatment guided by their molecular diagnosis (gabapentin and lacosamide). Also, diagnosed patients can avoid further diagnostic investigations such as EMGs, MRIs, and other tests.

### 3.6. Recommendations for Referrals from Pain Medicine to Medical Genetics

Individual genetic syndromes are rare, and clinicians must maintain a high degree of suspicion to make a diagnosis. Syndromes associated with chronic pain seen in Medical Genetics clinic include porphyria, hypophosphatasia, Ehlers-Danlos syndrome, and osteogenesis imperfecta. Importantly, many of these syndromes have a disease-specific targeted therapy (e.g., hematin for porphyria).

We recommend that Pain Medicine clinicians consider referring the following patients to Medical Genetics: (a) patients with a pain syndrome and family history of the same syndrome in 2 or more family members, (b) young patients with no explanation for their pain syndrome, (c) individuals of any age with very uncommon presentations, such as the rectal pain of PEPD, and (d) patients with a genetic disorder that may be contributing to their pain (porphyria). These recommendations are based on expert opinion. An online directory of genetic providers isavailable from the National Society of Genetic Counselors (http://www.nsgc.org/).

## Figures and Tables

**Table 1:** Clinical features, triggers, and mutations of sodium channel pain syndromes. Differences between small nerve fiber neuropathy, inherited erythromelalgia, and paroxysmal extreme pain disorder. ^*∗*^The mutations listed are meant to demonstrate phenotypic variability in single mutations; this is not an exhaustive list of mutations.

Pain syndromes	Small nerve fiber neuropathy (SFN)	Inherited erythromelalgia (IEM)	Paroxysmal extreme pain disorder (PEPD)
Clinical features	Cold, burning or electric-like pain, tingling or a pins-and-needles sensation, allodynia and hyperesthesia in feet, and distal extremities [10].	Burning pain, swelling, and skin redness in the distal extremities (feet and, less frequently, the hands). Some individuals have allodynia and hyperalgesia [19, 20].	Skin redness and warmth, severe pain in various parts of the body, typically in the lower part of the body, especially around the rectum, but also can be in head and face, especially the eyes and jaw [16]. Tonic nonepileptic seizures, flushing, watering of eyes or nose, hypersalivation, and weakness (hours to day) related to site of pain [17].

Triggers	None known [10].	Precipitated by mild warmth, standing, exercise, alcohol, and other vasodilating agents, relieved by cooling [16].	Trauma, childbirth, defecation, eating, taking medications, and cold [13].

Duration	Persistent [10].	Occurs multiple times per day and can become constant [4].	Seconds to hours [16].

SCN9A/Na_v_1.7^*∗*^	c.554G>A, p.R185H [14] c.684C>G, p.I228M [14] c.1867G>A, p.D623N [14] c.2159T>A, p.I720K [14] c.2215A>G, p.I739V [21] c.2794A>C, p.M932L [14] c.2971G>T, p.V991L [14] c.4596G>A, p.M1532I [14]	c.29A>G, p.Q10R [22, 23] p.L245V [24] c.406A>G, p.I136V [25] c.647T>C, p.F216S [19] c.721T>A, p.S241T [5] c.1185C>A, p.N395K [19] c.2468T>G, p.L823R [26] c.2543T>C, p.I848T [3] c.2573T>A, p.L858H [3] c.2572C>T, p.A863P [27] c.2623C>G, p.Q875E [28] c.4345T>G, p.F1449V [4]	c.2986C>T, p.R996C [13] c.3892G>T, p.V1298F [13] c.3893T>A, p.V1298D [13] c.3895G>T, p.V1299F [13] c.4382T>C, p.I1461T [13] p.F1462V [13] c.4391C>T, p.T1464I [13] G1607K [29] c.4835T>C, p.L1612P [30] c.4880T>A, p.M1627K [13]

SCN10A/Na_v_1.8^*∗*^	c.1661T>C, p.L554P [31] c.3910G>A, p.A1304T [31] c.4568G>A, p.C1523Y [31] c.4984G>A, p.G1662S [32] c.5116A>G, p.I1706V [33]		

SCN11A/Na_v_1.9^*∗*^	c.1142T>C, p.I381T [34] c.3473T>C, p.L1158P [34]		

**Table 2:** Small nerve fiber symptom inventory questionnaire: responses of the patient described in this report are marked with x's. The patient has some (0–3) symptoms of small fiber neuropathy.

	Symptoms of small nerve fiber neuropathy	Never	Sometimes	Often	Always
Autonomic	Sweating		x		
	Diarrhea		x		
	Constipation				x
	Urination problems	x			
	Dry eyes				x
	Dry mouth	x			
	Orthostatic complains	x			
	Palpitations	x			
	Hot flashes	x			

Sensory	Sensitive skin	x			
	Burning feet	x			
	Sheet intolerance	x			
	Restless legs	x			

**Table 3:** SCN9A-associated features questionnaire. The responses of the patient described in this case report are shown.

Have you ever had (including early childhood):	Yes/no	If yes, at what age did those features begin?	If yes, how often (sometimes, often, always)
Seizures	No		
Rectal pain	Yes	45-increased in severity	often
Constipation	Yes	3	always
Sensitivity to cold temperatures	Yes	51	always (likes ambient temperature of 27°C)
Redness and swelling of the hands or feet	Yes	36 (during pregnancy)	
Pain during/following tear secretion	No (pain when eyes dry)		
Pain during/following flushing	No		
Pain during/following sinus congestion	Yes	37	sometimes
Pain during/following defecation	Yes	45	always
Pain during/following eating	No		
Pain during/following strong emotions	Yes	53	often
